# Crisis Experience and Purpose in Life in Men and Women: The Mediating Effect of Gratitude and Fear of COVID-19

**DOI:** 10.3390/ijerph20156490

**Published:** 2023-08-01

**Authors:** Agnieszka Lasota

**Affiliations:** Institute of Psychology, Pedagogical University of Krakow, 30-084 Krakow, Poland; agnieszka.lasota@up.krakow.pl

**Keywords:** gratitude, fear of COVID-19, crisis experience, purpose in life, gender differences

## Abstract

Aim: This study investigated whether gratitude and fear of COVID-19 mediated the relationship between crisis experience during the pandemic and purpose in life in men and women. Methods: Six hundred and five participants aged between 18 and 60 years (M = 25.6; *SD* = 8.39) completed the Gratitude, Resentment, and Appreciation Scale—Short Form (GRAT-S), Fear of COVID-19 Scale, and Purpose in Life Test (PIL). In addition, the respondents were questioned about life crises during the previous six months (e.g., the death of a loved one or illness). Results: Men and women differed in their endorsement of gratitude or fear of COVID-19 as a mediator. Women’s experiences of the crisis related both directly and indirectly to purpose in life, with gratitude and fear of COVID-19 mediating this relationship. In men, only the indirect path, from crisis experience, through gratitude, to purpose in life, turned out to be significant. Conclusion: The results of this study suggest that coping styles differ in men and women and, as a consequence, that crisis interventions need to take gender into account.

## 1. Introduction

Mental health researchers have found that the COVID-19 pandemic has had a significant impact on mental, physical, and social health [1,2]. The pandemic has caused severe stress for the general population worldwide [3]. Many studies have suggested that the current situation will have a significant psychological effect into the future [4,5]. The COVID-19 pandemic has exacerbated mental health issues [4,6,7,8]. Worldwide, an increase in emotional, behavioral, physical, and mental health problems has been confirmed in all countries [9]. Some researchers [6,7] have found that traumatic experiences resulting from the COVID-19 pandemic (i.e., emotional crisis, loss of loved ones, and illness) are associated with psychological, social, and physical health issues.

People’s daily lives have changed, leading to fear and anxiety. This has affected their mental and physical well-being [10]. For this reason, it seems necessary to identify the factors that put individuals at risk of developing mental health difficulties and those that protect against them. Thus, this study aimed to provide more evidence on whether having experienced a crisis caused by COVID-19 affects the level of purpose in life. In addition, we wanted to determine if positive resources, such as gratitude, can help individuals to maintain their purpose in life, or whether a fear of COVID-19 can lower it. We expected gender differences based on previous scientific reports [11].

### 1.1. The Relationship between the Crisis Experience and Purpose in Life in the Pandemic Time

Research on life purpose, especially in difficult times such as the COVID-19 pandemic, helps us to understand the potential resources of people [12]. A sense of purpose in life can be understood as an individual’s subjective belief that their life has a deeper meaning and purpose [13]. Meaning in life is the strongest human motivator [14]. Purpose in life is positively related to good health, quality of life, and well-being [15,16]. Life satisfaction, hope, optimism, coping, and well-being can lead to an increase in one’s sense of purpose in life [17,18,19]. Having a purpose in life can also play a protective role against depression, feelings of hopelessness, poor mental health symptoms, and mental disorders [15,20,21,22]. The experience of a crisis in a person’s life can significantly reduce the sense of meaning in life. Individuals may experience a decreased sense of purpose caused by the loss of loved ones, job loss, social isolation, and feelings of anxiety and insecurity. The results of the study showed that during the COVID-19 pandemic, even people who did not declare a severe crisis felt sad and depressed, and consequently lost meaning and purpose in life [10,15]. Research confirms that people can experience grief not only after loss of a loved one but also after other life-changing losses, such as job loss or divorce [10]. Early evidence of the pandemic’s impact suggests that anxiety, high stress, and depressive symptoms were the most commonly reported emotional and mental health issues [23,24]. Many researchers emphasize that finding meaning in life plays a significant role in recovery from grief, suggesting that it may lead to reduced stress and post-traumatic growth [10,25]. Crises caused by COVID-19 can also trigger a deeper reflection on life and values, which allows for the redefinition, strengthening, or rediscovery of a sense of purpose. A few studies have shown a positive relationship between adversity and a sense of purpose in life [26]. According to the theory of logotherapy, the capacity of humanity is the ability to resist both one’s own physical and mental limitations, as well as the external influences of the situation. The outcome of this could be the pursuit or enhancement of a life purpose through self-reflection [14]. People understand that in difficult experiences they can discover new opportunities, and commitment to finding a purpose in life can help them to cope with adversity [14,27].

A sense of purpose has been identified by numerous researchers as a significant psychological resource [28]. Having a clear purpose in life is associated with reducing stress and negative emotions, and it facilitates emotional regeneration in the face of adversity [27,29]. People are physically and psychologically healthier when they have a purpose in life [28]. Therefore, because the effects of experiencing a crisis during a pandemic on mental health can be significant [30], finding a sense of meaning in life can be an essential element in the mental recovery process [31]. Research also suggests that, first, people may need cues to make sense in a structured way [32], and second, individual characteristics, such as the age and gender of the subjects, should be considered. Some studies have confirmed gender differences, where women have a higher sense of meaning in life than men [12,33]. Xi et al. [34] showed a difference in purpose in life in favor of women. The authors suggested gender differences can be explained by altruism. Women are more likely to display altruistic attitudes and behaviors associated with a higher purpose in life.

### 1.2. Crisis Experience, Gratitude, and Purpose in Life

According to several theories and studies, gratitude is the emotion that most effectively mitigates the negative effects of adversity [35]. When people are affected by misfortune, they appreciate more positive things in life, such as relationships with loved ones or material and intangible resources, which provokes them to feel gratitude [36]. Gratitude plays an important role in overcoming trauma despite intense suffering [37]. Being grateful helps us to cope with the stress, anxiety, and unpleasant consequences of COVID-19 [38]. Sometimes a crisis prompts individuals to reflect on their own lives, meaning, purpose, and plans for the future. Through self-reflection, people can redefine their life and develop mental resilience and better coping skills for adversity and post-traumatic growth. Wood et al. [39] believed that gratitude is part of a broader life orientation in which people appreciate the positive things that have happened to them. Some people appreciate each new day, notice the kindness and sacrifices of others, and are grateful for every positive moment in their lives. Such a positive life orientation can be contrasted with a depressive worldview, which is usually associated with focusing on the negative aspects of oneself, the world, and the future [40]. Jans-Beken [38] proposed that gratitude should be seen not only as a tendency to see positive benefits and prosperity but also as gratitude for adversity. Being grateful for negative events and people, illnesses, or losses is a more difficult part of gratitude. In difficult times, crises such as a pandemic, people often feel empty and anxious and lose control over their lives. When they realize their limitations, their sense of gratitude for their own lives is strengthened. People can benefit from a grateful outlook on life in times of crisis [41]. When individuals think about death, they confront it and appreciate their own lives more [42]. Mature gratitude, i.e., one that focuses on both the positive and negative aspects of life, helps deal with crises and difficult times [38]. Gratitude is a powerful predictor of subjective happiness, life satisfaction, and well-being [43,44]. Studies have confirmed that gratitude is positively associated with hope and optimism and negatively associated with anxiety, depression, and jealousy [45]. Grateful people have more positive traits [44,45] and positive views of their social environment [46]. They can focus on the positive aspects of their environment with a greater appreciation for their lives and possessions. They more often feel the impact of gratitude [45,47] and use productive coping strategies [44]. The findings of several studies [48] have confirmed that higher-order gratitude, comprising diverse components such as thanking others, thanking God, cherishing blessings, appreciating difficulties, and cherishing moments, explains the variability in subjective well-being, particularly positive affect and life satisfaction. Gratitude allows us to look more optimistically at life, our own experiences, relationships, and others’ behaviors [47,49]. It is a positive feeling that comes from appreciating and acknowledging the benefits we receive from other people or lives. Gratitude is positively linked to a sense of purpose and meaning in life [20,50,51]. Both a disposition towards gratitude and a sense of purpose in life may help to buffer stress-related psychological disturbances caused by COVID-19 [16]. The empirical evidence on differences in the experience of gratitude depending on gender in the vast majority of cases supports this thesis. Previous research shows that women are more likely to express gratitude for the little things in life, while men are less likely to feel and express gratitude and make more critical judgments of gratitude [43,52,53].

### 1.3. The Crisis Experience, Fear of COVID-19, and Purpose in Life

The COVID-19 outbreak has been one of the biggest crises in the world in recent times, and fear is associated with a reduction in people’s quality of life [1]. Research has confirmed that fear of COVID-19 negatively affects all aspects of life in the general population [11,54,55]. The pandemic disrupted daily routines, led to economic instability, and created uncertainty regarding the future. This lack of control and uncertainty may have exacerbated COVID-19 concerns, as individuals may have felt powerless to face the crisis and waited for it to end. Uncertainty related to the experience of epidemics in the world still evokes fear and a sense of unpredictability [56,57]. High fear of coronavirus is a predictor of mental stress, depression, and even PTSD [58,59]. Moreover, a pandemic and the experience of the crisis may disrupt or undermine a society’s personal goals and plans. This can lead to a sense of confusion, loss of direction in life, and reduced motivation, consequently reducing the purpose and sense of meaning in life. Fear of COVID-19 has been found to have a negative correlation with resilience, life satisfaction, and well-being [57,60,61]. Additionally, research conducted during the pandemic has revealed strong links between gender and the fear of COVID-19. It has been shown in numerous studies, especially during the first wave of the pandemic, that women linked the coronavirus with a greater health risk than men [11,62]. A study conducted in 59 countries found the pandemic had a more pronounced impact on women. They reported lower expectations about the health consequences of COVID-19 and a greater fear of death, which can be considered a significant predictor of anxiety symptoms [63]. However, other researchers [64] found no gender differences in the fear of COVID-19. Subsequent studies have shown contradictory results. For example, some studies have shown that men have a greater fear of COVID-19 than women [65,66]. Other studies have shown no gender differences [67]. A meta-analysis by Metin et al. [11] confirmed that gender differences may depend on place of residence or culture, since, for example, studies conducted in Europe more often confirmed that the level of fear of COVID-19 was higher in women [68,69]. Studies conducted in Middle Eastern countries, however, showed the opposite relationship, with men having a greater fear of COVID-19 [65,66]. Research examining gender differences in the fear of COVID-19 revealed inconsistent findings, so further investigation was needed.

### 1.4. The Present Study

This study aims to examine the relationship between crisis and purpose in life. Based on the reviewed theory [14,27,38,52] and research [11,50,70], we examined whether gratitude and fear of COVID-19 mediate the association between the experience of crisis and having a purpose in life. We also explored the potential moderating effect of gender on this relationship. The hypotheses were as follows: H1. Crisis experiences have a direct effect on purpose in life (PIL). H2. Gratitude mediates the relationship between crisis experience and PIL. H3. Fear of COVID-19 mediates the link between crisis experience and purpose in life. H4. Gender moderates the relationship between (a) gratitude and PIL; (b) fear of COVID-19 and PIL; (c) crisis experience and PIL.

## 2. Materials and Methods

### 2.1. Participants and Procedure

This study included 605 adults from Poland (444 females and 161 males). The respondents were between 18 and 60 years old (M = 25.6; *SD* = 8.39). Most of them (2/3) had secondary education, 32% had higher education, 2% had only completed primary education, and 2% had completed vocational education. The majority of the respondents lived in large cities (60%, n = 366), less in the country (25%, n = 146) and in small towns (15%, n = 93). Data on work activity showed that students made up the largest percentage of the respondents (44%); 27% were adults working and learning or studying, and 24% of the respondents were actively working. Another 5% declared that they were neither working nor studying.

Data were collected between September and December 2021. We used an online questionnaire distributed via social networking sites. All the respondents provided informed consent online. Ethical procedures were followed throughout the study. Before data collection, respondents were assured of anonymity.

### 2.2. Methods

The online survey contained psychological tools and demographic questions designed to collect personal data from Polish adults. The demographic information collected included age, gender, level of education, work activity, and place of residence. 

#### 2.2.1. Crisis Experience

Participants answered the following question: Have you experienced a crisis related to the COVID-19 pandemic in the last six months? The respondents answered yes or no. By marking the answer yes, they had many options to choose from regarding the type of crisis (e.g., death of a loved one, illness, loss of job, deterioration of family relations). They also could describe a situation they considered a crisis experience.

#### 2.2.2. Gratitude

Gratitude was measured using the Gratitude, Resentment, and Appreciation Scale—Short Form [43] in the Polish adaptation [71]. The GRAT-S is a multidimensional tool designed to measure dispositional gratitude. It comprises three subscales: simple appreciation (SA), lack of a sense of deprivation (LOSD), and appreciation for others (AO). Participants rated their responses to 16 items on a 9-point Likert scale (e.g., I feel deeply appreciative for the things others have done for me in my life). In this study, the internal reliability was 0.73.

#### 2.2.3. Fear of COVID-19

The Fear of COVID-19 Scale [72] in the Polish translation was used to examine the fear of COVID-19. This 7-item unidimensional scale uses a 5-point Likert scale ranging from 1 (strongly disagree) to 5 (strongly agree) (e.g., I am afraid of losing my life because of Coronavirus). The composite score ranged from 7 to 35, with a higher score indicating a greater fear of COVID-19. The reliability was α = 0.86.

#### 2.2.4. Purpose in Life

Purpose in life was assessed using the brief 6-item Polish version [73] of the Purpose in Life Test [74]. Each item is rated on a five-point scale that was specifically designed (e.g., Every day is …, 0 = the same to 5 = new). The short version of the scale has demonstrated good validity. The internal consistency coefficient was 0.92.

### 2.3. Data Analysis

Before the main analysis, the data were screened for potential errors in the predicted range of values and indicators of careless answers. The Mahalanobis distance was used to evaluate the outliers. All observations (N = 605) were included in the statistical analyses. Statistical analyses were conducted using the IBM SPSS software (version 28, PS IMAGO PRO 8.0, Predictive Solutions). Means, standard deviations, and normality checks were performed for all main study variables. The examined variables were standardized using SPSS after descriptive statistics. An independent Welch’s *t*-test was used to compare the differences between the female and male samples. Pearson’s correlations were used to assess the associations between the four analyzed variables: crisis, gratitude, fear of COVID-19, and purpose in life. A moderated mediating analysis (model 15) and two parallel mediating analyses (model 4) were estimated using the PROCESS macro for SPSS version 4.2 [75]. The bootstrapped samples were set to 5000 at 95% bias-corrected confidence intervals.

## 3. Results

### 3.1. Preliminary Analyses

The results of the descriptive statistics, gender differences, and the bivariate correlation coefficients of crisis, purpose in life, gratitude, and fear of COVID-19 are reported in Table 1. An independent sample Welch’s *t*-test revealed gender differences for most of the examined variables. The results indicated that women, compared to men, had higher levels of gratitude but also higher levels of fear of COVID-19. Women also reported experiencing a crisis caused by the pandemic more often than men did. The respondents did not differ in their level of purpose in life. Correlation analyses indicated that, in the female sample, crisis experience was significantly and negatively linked with purpose in life and gratitude, and positively correlated with fear of COVID-19. Purpose in life was strongly positively correlated with gratitude and weakly negatively correlated with fear of COVID-19. Among men, no significant relationship was found between crisis and purpose in life. Moreover, in the male sample, fear of coronavirus was only positively related to the crisis experience but was not related to purpose in life or gratitude.

### 3.2. Moderated Mediation Analysis

**Figure 1:**
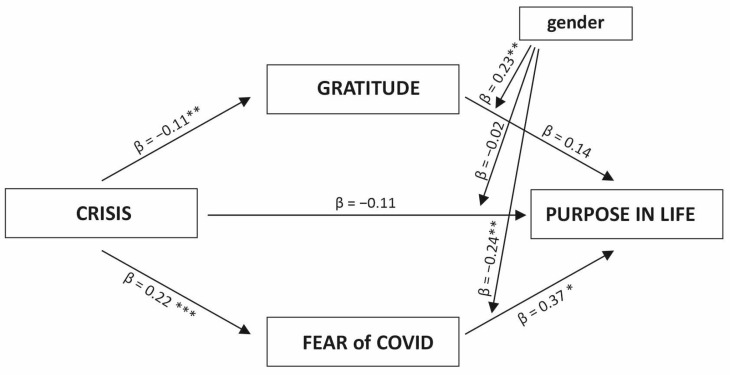
Parallel moderated mediation model of the crisis experience on purpose in life using gratitude and fear of COVID-19 as mediators. Note: standardized coefficients are presented. *** *p* < 0.001, ** *p* < 0.01, * *p* < 0.05.

The moderated mediation analysis is illustrated in Figure 1. A crisis is a predictor variable, gratitude and fear of COVID-19 are mediator variables, and purpose in life is an outcome variable. Gender is a moderator of the relationship between the predictor and mediators and the outcome variable (model 15 in PROCESS).

**Figure 2:**
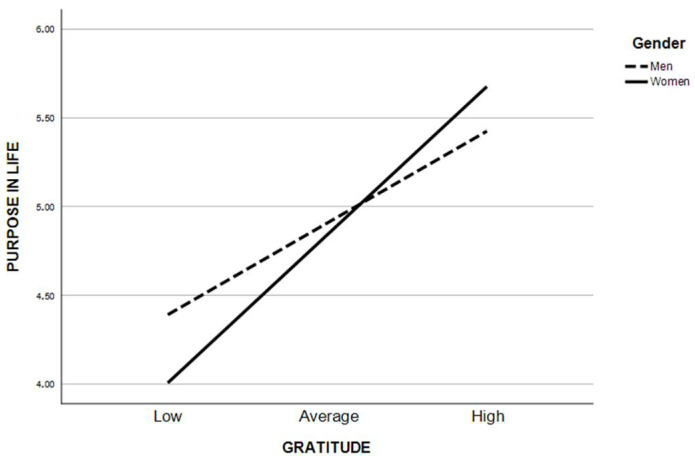
A visual representation of the moderating effect of gender on the association of gratitude with purpose in life.

Crisis experience was found to be a significant negative predictor of gratitude (b = −0.19, SE = 0.107, t = −2.75, *p* < 0.01, CI [−0.34, −0.06]). In addition, crisis was positively related to the fear of COVID-19 (b = 0.32, SE = 0.06, t = 5.42, *p* < 0.001, CI [0.21, 0.44]). The direct relationship between crisis experience and purpose in life was not significant (b = −0.11, SE = 0.41, t = −0.28, *p* =.78, CI [−0.92, 0.69]). In addition, the interaction between gender and crisis was statistically insignificant (b = −0.06, SE = 0.23, t = −0.27, *p* = 0.78, CI [−0.51, 0.39]). Gratitude was not directly related to purpose in life (b = 0.23, SE = 0.21, t = 1.10, *p* = 0.27, CI [−0.92, 0.69]), but the gender interaction with gratitude was significant (b = 0.36, SE = 0.12, t = 3.05, *p* < 0.01, CI [0.13, 0.59]). This model for purpose in life was a good fit for the data (ΔR^2^ = 0.31, *F*(7,597) = 38.80, *p* < 0.001). Gender was a significant moderator of the relationship between gratitude and purpose in life, and increased the explained variance (ΔR^2^chng = 0.010, *F*(1,597) = 9.33, *p* < 0.01). This visualization is shown in Figure 2. The effect is stronger in the group of women (b = 0.95, SE = 0.07, t = 14.20, *p* < 0.000, CI [0.82, 1.08]) compared to men (b = 0.59, SE = 0.10, t = 6.03, *p* < 0.000, CI [0.40, 0.78]).

**Figure 3:**
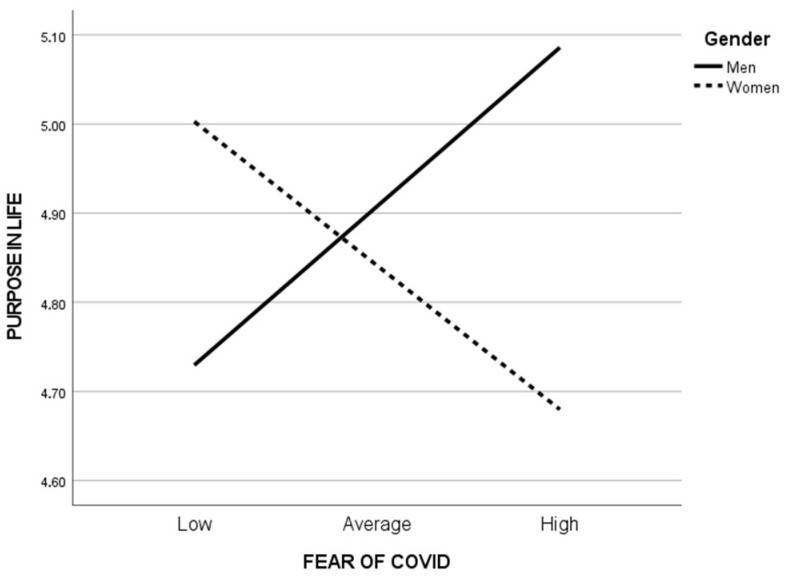
A visual representation of the moderating effect of gender on the association of fear of COVID-19 with purpose in life.

Fear of COVID-19 was significantly related to purpose in life (b = 0.69, SE = 0.28, t = 2.43, *p* < 0.05, CI [0.13, 1.26]) and the interaction between gender and the mediator was significant (b = −0.45, SE = 0.16, t = −2.92, *p* < 0.01, CI [−0.76, −0.15]). Gender was a significant moderator of the relationship between fear of COVID-19 and PIL and increased the explained variance (ΔR^2^chng = 0.010, *F*(1,597) = 8.48, *p* < 0.01). This visualization is illustrated in Figure 3. Moderation analysis showed that the effect was significant in the female group (b = −0.22, SE = 0.07, t = −2.91, *p* < 0.01, CI [−0.36, −0.07]), but insignificant in the male group (b = 0.24, SE = 0.14, t = 1.73, *p* = 0.08, CI [−0.03, 0.51]).

### 3.3. Parallel Mediation Separately for Women and Men

Two separate multiple mediation analyses were conducted to explore differences between men and women in whether gratitude and fear of COVID-19 mediated the relationship between crisis experience and purpose in life (model 4 in PROCESS).

**Figure 4:**
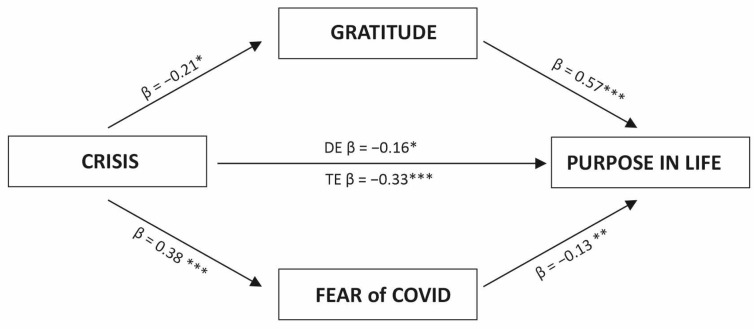
Parallel mediation model of the crisis experience on purpose in life using gratitude and fear of COVID-19 as mediators in the female sample (n = 444). Note: standardized coefficients are presented. *** *p* < 0.001, ** *p* < 0.01, * *p* < 0.05.

In women (Figure 4), the crisis was a significant negative predictor of gratitude (b = −0.17, SE = 0.08, t = −2.15, *p* < 0.05, CI [−0.33, −0.01]) and a positive predictor of fear of COVID-19 (b = 0.28, SE = 0.07, t = 3.94, *p* < 0.001, CI [0.14, 0.42]). Women’s crisis experience was also directly related to their purpose in life (DE b = −0.23, SE = 0.11, t = −2.04, *p* < 0.05, CI [−0.44, −0.01]). These results indicated that both mediators were significantly associated with PIL. Gratitude was positively related to purpose in life (b = 0.95, SE = 0.06, t = 14.9, *p* < 0.001, CI [0.82, 1.32]), whereas fear of COVID-19 negatively correlated with PIL (b = −0.23, SE = 0.07, t = −3.25, *p* < 0.01, CI [−0.37, −0.09]). The model showed a good fit with the data (ΔR^2^ = 0.36, *F* = 83.11, *p* < 0.001). The total effect of crisis on purpose in life (b = −0.46, SE = 0.13, t = −3.42, *p* < 0.001, CI [−0.72, −0.19]; ΔR^2^ = 0.026, *F*(1,441) = 11.74, *p* < 0.001). The indirect effect of the crisis experience on purpose in life via gratitude was significant (b = −0.17, β = −0.12, SE = 0.05, CI [−0.22, −0.01]), accounting for 36% of the total effect. Fear of COVID-19 was also a significant mediator in the female sample (b = −0.07, β = −0.05, SE = 0.02, CI [−0.09, −0.01]). The indirect effect accounted for 14% of the total effect.

**Figure 5:**
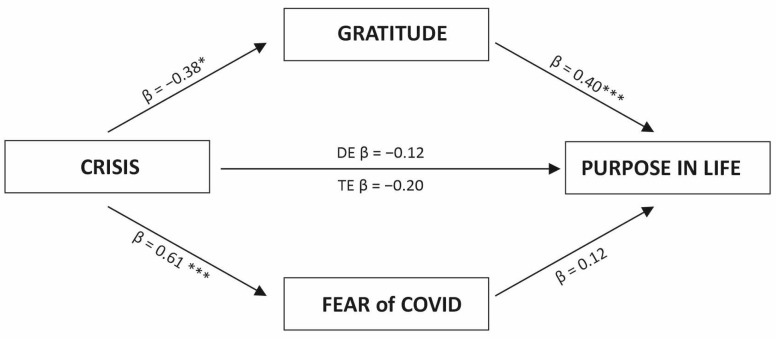
Parallel mediation model of the crisis experience on purpose in life using gratitude and fear of COVID-19 as mediators in the male sample. Note: standardized coefficients are presented. *** *p* < 0.001, ** *p* < 0.01, * *p* < 0.05.

The analysis of the results among men (Figure 5) showed that crisis was linked to both mediators, showing a strong positive correlation with fear of COVID-19 (b = 0.43, SE = 0.10, t = 4.06, *p* < 0.001, CI [0.22, 0.64]) and an inverse link with gratitude (b = −0.37, SE = 0.15, t = −2.48, *p* < 0.05, CI [−0.67, −0.017]). The direct effect of crisis on purpose in life was not significant (b = −0.12, SE = 0.22, t = −0.80, *p* = 0.42, CI [−0.61, 0.26]). Only gratitude was significantly related to purpose in life (b = 0.59, SE = 0.11, t = 5.37, *p* < 0.001, CI [0.37, 0.80]). The fear of COVID-19 was not significantly associated with PIL (b = 0.24, SE = 0.15, t = 1.55, *p* = 0.12, CI [−0.06, 0.54]). The model showed a good fit with the data (ΔR^2^ = 0.18, F = 11.71, *p* < 0.001). The total effect was insignificant (b = −0.29, SE = 0.22, t = −1.30, *p* = 0.19, CI [−0.74, 0.15]; ΔR^2^ = 0.010, *F*(1,159) = 1.69, *p* = 0.19). The standardized indirect effect (IE) of the crisis experience on PIL via gratitude was found to be significant because the 95% confidence interval did not include zero (β = −0.15, SE = 0.07, CI [−0.30, −0.03]); however, via fear of COVID-19 was insignificant (β = 0.07, SE = 0.05, CI [−0.02, 0.19]).

## 4. Discussion

In this study, a moderated mediation model was proposed, in which gratitude and fear of COVID-19 mediated the relationship between crisis experience and purpose in life. We also postulated that gender may moderate the relationship between the variables. 

As hypothesized, our findings show gender differences. Women presented a higher fear of COVID-19 and more often declared experiencing a crisis caused by COVID-19 than men. These results are consistent with those obtained previously, particularly in European countries [11]. Previous research has demonstrated higher perceptions of COVID-related health and social risks by women in individualistic societies, their greater awareness of danger, and maladjustment in the face of threats [67,68,69]. This study also revealed gender differences in gratitude. This is in line with previous studies showing that women have a higher level of gratitude than men [52,53]. Gender turned out to be an important moderator between the mediators (gratitude and fear of COVID-19) and the explained variable—purpose in life. In both men and women, gratitude was associated with a higher purpose in life, and this effect was stronger in women. The higher the level of gratitude expressed by women, the more often they reported having a purpose in life. A low level of gratitude, undervaluing what one has received in life, was associated with a lower or lack of purpose in life. According to Frankl’s [14] logotherapy theory, PIL is not only an internal psychological resource that can be drawn upon in times of crisis but also a positive construct that can be fostered among people through various paths [28,52]. These findings show that gender moderated the relationship between fear of COVID-19 and purpose in life. This effect was significant among the women. The higher the fear of coronavirus infection, the lower the purpose in life. This effect was not statistically significant in men. The results of our research may contribute to expanding the existing knowledge about female perception of the COVID-19 risk and of this pandemic as being more dangerous for the population [11,76]. In addition, women express their emotions more easily, whereas men tend to suppress them and seem stronger [77].

Using separate mediation analyses based on the respondents’ gender allowed us to establish additional findings that partially supported our hypotheses about gratitude and fear of COVID-19 as mediators in the relationship between crisis and PIL. In women, experiencing a crisis was associated both directly, with lowering their purpose in life, and indirectly, through the fear of contracting COVID-19. Gratitude also turned out to be an important mediator, but one that strengthened the sense of purpose in life despite experiencing difficult crises during the pandemic. The results suggest that the crisis experience played a triggering role, exacerbating the fear of COVID-19 in women more than in men [11], leading to a loss of purpose in life [15]. Psychological support with an emphasis on meaning in life might be most helpful for women [15].

The mediation analysis conducted among men showed a slightly different relationship profile between variables. The experience of a crisis by men during the COVID-19 pandemic was more strongly linked to fear of infection. Crisis experience in men was also more negatively associated with expressing gratitude. Men who experienced difficult situations were less willing to express their gratitude. In addition, the link between the crisis experience and PIL was statistically insignificant. Thus, the first hypothesis was not confirmed in the male group. In men, difficult experiences do not directly reduce the sense of purpose in life. Additionally, fear of COVID-19 was not directly related to the purpose in life among men. Only the indirect path, from crisis experience, through gratitude, to purpose in life, was significant. The crisis experience lowered the sense of gratitude; however, gratitude increased purpose in life. Hence, the hypotheses about mediation through gratitude and fear of COVID-19 on PIL were confirmed partially. These findings support previous research [36]. The results of Xi et al. [34] are consistent with the idea that altruistic activities help men to find a purpose in life. Our results also reinforce previous findings indicating that positive resources can improve mental health during a pandemic [70].

The results suggest that gender may be a significant factor in developing intervention programs, supports, and methods for adults going through a crisis. COVID-19 has highlighted the fragility of mental health during difficult times, and practical solutions are needed to manage it.

### 4.1. Practical Implications

This research suggests that experiencing difficult crises in men is significantly associated with a decrease in positive resources (such as gratitude) and an increase in negative resources (such as increased fear of illness), but not with purpose in life. For women, experiencing a crisis significantly reduced their purpose. Psychological therapies aimed at reinterpreting past difficult experiences and reclaiming purposes in life can reduce the negative psychological effects of the pandemic [4]. Counterfactual thinking, which can mitigate stressful or traumatic experiences, can be one such method [3,78]. Another example of support could be life-creation interventions that offer a way to help individuals find new meaning in life [10,79]. Finally, it is worth using programs and activities that develop gratitude as an attitude or strength of character.

### 4.2. Limitations

This study has some limitations. Cross-sectional studies using self-reported questionnaires prevented us from determining the direction of the impact of the variables. We have a homogeneous sample of respondents (adult Poles with secondary and higher education living mainly in large cities). Therefore, the results cannot be generalized to other cultures or nationalities. Lack of control for situational factors related to crises (i.e., the duration of the crisis) is also a limitation of this study. Finally, future research examining the association between crises and purpose in life may apply alternative models, including other mediators. Perhaps we should also consider another model of mediation, where purpose in life would be the explained variable and crisis experience would be the mediator, preceded by output variables, such as gratitude or fear of COVID-19. Furthermore, the type of crisis experienced by the surveyed adults (death of a loved one, illness) should have been considered, checking whether it is significant in this relationship. The conduct of path analysis may reveal a more complex mechanism of the link between crisis experience and purpose in life than that presented in this article.

## 5. Conclusions

The results of this study highlight the need to create different prevention and intervention programs depending on the gender of clients or patients. Men and women may use different emotional strategies to cope with very stressful situations. These findings may be useful in developing specialized psychological support, especially during crises.

## Figures and Tables

**Table 1:** Gender differences and bivariate correlations between crisis experience, purpose in life, gratitude, and fear of COVID-19 in the female and male samples.

Variables	Sex	M	*SD*	t	/d/	2.	3.	4.
1. Crisis Experience	F	1.62	0.48	2.66 **	0.25	−0.16 **	−0.10 *	0.19 **
	M	1.50	0.50			−0.10	−0.19 *	0.31 **
2. Purpose in Life	F	4.90	1.38	1.06	0.10		0.58 **	−0.14 **
	M	4.76	1.43				0.41 **	0.11
3. Gratitude	F	6.39	0.83	3.79 ***	0.35			−0.00
	M	6.10	0.96					0.03
4. Fear of COVID-19	F	1.78	0.75	2.27 *	0.21			
	M	1.62	0.70					

Note: M = mean, *SD* = standard deviation, t = Welch’s t-statistic, /d/ = effect size Cohen’s d, *** *p* < 0.001, ** *p* < 0.01, * *p* < 0.05.

## Data Availability

The dataset presented in this study is available on reasonable request from the corresponding author.
